# Supplementary material for: Modeling glioblastoma heterogeneity as a dynamic network of cell states
Source: Mol Syst Biol. 2021 Sep 16;17(9):e10105. doi: 10.15252/msb.202010105 (PMC8444284; doi:10.15252/msb.202010105)
Supplement: Supplementary file 6 — Source Data for Figure 5 [file MSB-17-e10105-s004.zip › Figure5A_sourcedata/GSEA_3017/nosigdb_stateD.GseaPreranked.1622795010397/gsea_report_for_na_pos_1622795010397.html]

Report for na\_pos 1622795010397 [GSEA]

| GS  follow link to MSigDB | GS DETAILS | SIZE | ES | NES | NOM p-val | FDR q-val | FWER p-val | RANK AT MAX | LEADING EDGE || 1 | RICHARDS\_NOWAKOWSKI\_SCIENCE\_2017\_IPC\_NEN3\_UPREG | Details ... | 55 | 0.32 | 2.88 | 0.000 | 0.000 | 0.000 | 158 | tags=44%, list=12%, signal=48% |
| 2 | RICHARDS\_NOWAKOWSKI\_SCIENCE\_2017\_NIN2\_UPREG | Details ... | 78 | 0.18 | 1.85 | 0.000 | 0.054 | 0.074 | 290 | tags=45%, list=22%, signal=54% |
| 3 | NB\_KI\_CLUSTER7 | Details ... | 19 | 0.31 | 1.58 | 0.036 | 0.178 | 0.328 | 569 | tags=79%, list=43%, signal=137% |
| 4 | LARSSON\_STATE\_1 | Details ... | 28 | 0.24 | 1.53 | 0.033 | 0.160 | 0.385 | 155 | tags=43%, list=12%, signal=48% |
| 5 | DOROSZKO\_U3054MG\_CLUSTER\_8 | Details ... | 95 | 0.14 | 1.30 | 0.000 | 0.379 | 0.740 | 961 | tags=92%, list=73%, signal=312% |
| 6 | NB\_KI\_CLUSTER6 | Details ... | 56 | 0.14 | 1.30 | 0.136 | 0.319 | 0.741 | 762 | tags=79%, list=58%, signal=178% |
| 7 | RICHARDS\_NOWAKOWSKI\_SCIENCE\_2017\_NIN4\_UPREG | Details ... | 28 | 0.19 | 1.24 | 0.214 | 0.350 | 0.818 | 116 | tags=29%, list=9%, signal=31% |
| 8 | RICHARDS\_NOWAKOWSKI\_SCIENCE\_2017\_IPC\_NEN1\_UPREG | Details ... | 56 | 0.13 | 1.19 | 0.135 | 0.378 | 0.884 | 132 | tags=23%, list=10%, signal=25% |
| 9 | RICHARDS\_NOWAKOWSKI\_SCIENCE\_2017\_IN\_CTX\_CGE1\_UPREG | Details ... | 51 | 0.11 | 1.06 | 0.380 | 0.550 | 0.966 | 254 | tags=35%, list=19%, signal=42% |
| 10 | RICHARDS\_NOWAKOWSKI\_SCIENCE\_2017\_NIN5\_UPREG | Details ... | 48 | 0.12 | 1.05 | 0.333 | 0.511 | 0.970 | 988 | tags=92%, list=75%, signal=350% |
| 11 | DOROSZKO\_U3054MG\_CLUSTER\_0 | Details ... | 19 | 0.18 | 0.97 | 0.440 | 0.605 | 0.991 | 10 | tags=11%, list=1%, signal=10% |
| 12 | RICHARDS\_NOWAKOWSKI\_SCIENCE\_2017\_IPC\_NEN1\_UPREG\_1 | Details ... | 31 | 0.13 | 0.86 | 0.667 | 0.762 | 0.997 | 120 | tags=23%, list=9%, signal=24% |
| 13 | RICHARDS\_NOWAKOWSKI\_SCIENCE\_2017\_NIN3\_UPREG | Details ... | 24 | 0.13 | 0.80 | 0.758 | 0.808 | 0.998 | 116 | tags=21%, list=9%, signal=22% |
| 14 | RICHARDS\_NOWAKOWSKI\_SCIENCE\_2017\_U4\_UPREG | Details ... | 191 | 0.05 | 0.55 | 1.000 | 0.986 | 1.000 | 99 | tags=11%, list=7%, signal=10% |
Table: Gene sets enriched in phenotype **na **[plain text format]****
